# Immunogenicity and Safety of Biological E’s Monovalent rDNA Hepatitis B Vaccine (BEVAC^®^) in Neonates and Infants: A Multicentre, Randomized, Phase IV Non-Inferiority Trial

**DOI:** 10.3390/v18040472

**Published:** 2026-04-17

**Authors:** Subhash Thuluva, Subbareddy Gunneri, Siddalingaiah Ningaiah, Vijay Yerroju, Rammohan Reddy Mogulla, Chirag Dhar, Kamal Thammireddy, Raju Esanakarra, Pradeep Nanjappa, Niranjana S. Mahantshetti

**Affiliations:** 1 Biological E Limited, Hyderabad 500 020, India; 2 Department of Pediatrics, Cheluvamba Hospital, Mysore Medical College & Research Institute, Mysore 570 001, India; 3 Department of Pediatrics, KLES Dr. Prabhakar Kore Hospital & Medical Research Centre, Belagavi 590 010, India

**Keywords:** hepatitis B vaccine, BEVAC^®^, neonates, immunogenicity, safety, randomized trial, non-inferiority, phase IV

## Abstract

Biological E’s BEVAC^®^ is a recombinant DNA hepatitis B vaccine that has been used in India for more than a decade in routine early-life immunization and has recently been prequalified by the World Health Organization (WHO). This multicentre, single-blind, parallel-group, randomized phase IV trial, conducted at seven study sites in India, compared the immunogenicity and safety of BEVAC^®^ with a licensed comparator vaccine (GeneVac-B^®^, Serum Institute of India Pvt. Ltd, Pune, India.) in healthy term neonates and infants. Participants received three 0.5 mL doses administered intramuscularly at birth (within 24 h), 6 weeks of age, and 14 weeks of age. The primary endpoint was seroprotection (anti-HBs IgG ≥10 mIU/mL) at 28 days after the third dose (Day 126), compared using a non-inferiority margin of −10%. Secondary endpoints included safety and tolerability outcomes through Day 126. A total of 468 neonates were randomized (234 per group), of whom 44% were female. At Day 126, seroprotection rates were 98.2% (95% CI: 95.39, 99.50) with BEVAC^®^ and 99.1% (95% CI: 96.78, 99.89) with the comparator; the between-group difference was −0.9% (95% CI: −3.09, 1.24), meeting the prespecified non-inferiority criterion. Solicited adverse events within 7 days after any dose occurred in 29.1% (95% CI: 23.3, 35.3) of BEVAC^®^ recipients and 35.0% (95% CI: 28.9, 41.5) of comparator recipients, most commonly pyrexia, injection-site pain, and swelling; all were mild-to-moderate. No serious adverse events were reported. BEVAC^®^ demonstrated non-inferior immunogenicity to the licensed comparator and a comparable safety profile.

## 1. Introduction

Hepatitis B virus (HBV) infection remains a major global public health problem, with an estimated 254 million people living with chronic HBV infection in 2022 [1,2]. Despite the availability of effective vaccines, HBV continues to drive a substantial burden of cirrhosis and hepatocellular carcinoma, contributing to approximately 1.1 million deaths annually [1,2]. HBV is transmitted through exposure to infected blood and body fluids; importantly, mother-to-child transmission (MTCT) at or around the time of birth remains a critical route of infection, particularly in high-burden settings [1,2]. The risk of chronicity is strongly age-dependent: up to 90% of neonates infected perinatally develop chronic HBV infection in the absence of timely prophylaxis, compared with far lower rates in adults [1,3]. Preventing mother-to-child transmission is therefore central to HBV elimination strategies.

To reduce perinatal transmission and its lifelong consequences, the World Health Organization (WHO) recommends administration of a timely hepatitis B vaccine birth dose, preferably within 24 h of birth, followed by completion of the infant hepatitis B vaccine series [1,4]. The birth dose is a cornerstone intervention, providing early protection during a period of high vulnerability and helping to prevent infection from maternal and early household exposure [1,5,6]. When implemented effectively alongside additional preventive measures where indicated, this strategy substantially reduces early-life HBV acquisition and the subsequent pool of chronic infection [1,6,7].

Recombinant DNA (rDNA) hepatitis B vaccines have been used for several decades and have an established efficacy and safety profile. In healthy infants, rDNA vaccines induce seroprotective immunity, typically defined as anti-HBs antibody concentrations ≥10 mIU/mL, in more than 95% of recipients when administered according to recommended schedules [4,8]. Global adoption of infant hepatitis B immunization has led to major declines in HBV infection and related disease; however, vaccination coverage remains uneven. WHO/UNICEF estimates indicate that global coverage of the hepatitis B birth dose remains suboptimal, and completion of the full infant series continues to fall short of targets in several regions [9]. In India, hepatitis B vaccination has been incorporated into the Universal Immunization Programme (UIP), with a birth dose recommended particularly for facility births; nevertheless, strengthening timely birth-dose delivery remains important for ensuring protection from the outset of life [10,11].

Several recombinant DNA hepatitis B vaccines are licensed for use in India, with GeneVac-B^®^ from the Serum Institute of India in Pune [12] and BEVAC^®^ from Biological E in Hyderabad among the most widely used. Both these vaccines are WHO-prequalified. To better understand how the vaccine performs in routine program settings in neonates and infants, a multicentre, phase IV, randomized non-inferiority trial comparing BEVAC^®^ with GeneVac-B^®^ in infants vaccinated according to the recommended schedule was conducted.

Here, we report findings from the phase IV trial. The primary objective was to evaluate immunogenicity, assessed by the proportion of infants achieving seroprotective anti-HBs concentrations following the primary series, alongside antibody titres (geometric mean concentrations and fold rise). Safety and tolerability were assessed through solicited local and systemic reactogenicity and monitoring of adverse events, including serious and medically attended events.

## 2. Materials and Methods

### 2.1. Study Design and Setting

This phase IV clinical trial was a multicentre, randomized, single-blind, active-controlled study conducted at 7 study sites in India. The trial was performed in accordance with the Declaration of Helsinki and International Council for Harmonisation Good Clinical Practice (ICH-GCP) guidelines. The protocol, informed consent form, and other relevant study documents were reviewed and approved by the Institutional Ethics Committees (IECs) at all study sites prior to study initiation. Written informed consent was obtained from the parent(s) or legally acceptable representative(s) of each participant before enrolment and prior to any study-related procedures. A list of clinical study sites, together with Ethics Committee approval dates, is included in the Institutional Review Board Statement.

This clinical trial was prospectively registered in the Clinical Trials Registry—India (CTRI; registration number: CTRI/2020/05/025012).

### 2.2. Participants

Healthy term neonates (≥37 weeks gestation) aged <24 h at enrolment, with birth weight ≥2500 g, were eligible for inclusion. Key exclusion criteria included preterm birth and/or low birth weight (<2500 g), any clinically significant medical condition or congenital disorder that could interfere with vaccine response or safety evaluation, known hypersensitivity to any vaccine component, and any contraindication to intramuscular injection. Neonates born to mothers known to be hepatitis B surface antigen (HBsAg)-positive were not enrolled, as such infants require hepatitis B immune globulin (HBIG) and a prevention regimen outside the scope of this trial [3]. Neonates who had received any hepatitis B vaccine prior to enrolment (e.g., birth dose administered outside the study) were excluded. The full inclusion and exclusion criteria are provided in the Appendix A.

### 2.3. Randomization and Blinding

Eligible participants were randomized in a 1:1 ratio to receive either BEVAC^®^ or the comparator vaccine (GeneVac-B^®^). Randomization was stratified by site and generated using an Interactive Web-based Response System (IWRS). Allocation concealment was ensured by dispensing sequentially numbered vaccine vials/labels corresponding to the randomization code.

The study was single-blind, with participants’ parents/legal representatives unaware of the assigned vaccine. The laboratory personnel involved in serology testing remained blinded to the treatment, and codes were used to link the participants to samples without revealing treatment assignment.

### 2.4. Vaccines and Immunization Schedule

BEVAC^®^ (Biological E. Limited, Hyderabad, India) is a recombinant DNA hepatitis B vaccine containing 10 µg of purified hepatitis B surface antigen (HBsAg) adsorbed onto aluminium hydroxide per 0.5 mL paediatric dose. GeneVac-B^®^ (Serum Institute of India Pvt. Ltd., Pune, India) similarly contains 10 µg recombinant HBsAg adsorbed onto aluminium hydroxide per 0.5 mL dose.

Study vaccines were stored and handled at 2–8 °C in accordance with manufacturer recommendations. Each participant received three doses of the assigned vaccine at 0, 6, and 14 weeks of age. The birth dose (week 0) was administered as soon as possible after birth, within 24 h of life. Subsequent doses were administered at approximately 6 and 14 weeks of age, with an allowable window of +7 days for the second and third doses. Vaccines were administered intramuscularly (0.5 mL) into the anterolateral thigh (vastus lateralis).

### 2.5. Concomitant Vaccination

Concomitant routine immunizations were administered in accordance with national immunization guidelines. Bacillus Calmette–Guérin (BCG) vaccine and oral polio vaccine (OPV) were administered at birth, where applicable, at a separate anatomical site from the hepatitis B vaccine. At 6, 10, and 14 weeks, participants received recommended age-appropriate vaccines (quadrivalent Diphtheria–tetanus–whole cell pertussis–H. influenzae type b, poliovirus, and rotavirus vaccines) as per the immunization schedule. No additional hepatitis B-containing vaccine outside the study protocol was administered. To avoid additional hepatitis B antigen exposure, a licensed quadrivalent vaccine containing Diphtheria–tetanus–whole cell pertussis–H. influenzae type b antigens (DTwP-Hib) was administered at 6–10–14 weeks.

### 2.6. Immunogenicity Assessments

Blood samples (~3 mL) were collected at baseline (prior to administration of the birth dose) and at 28 days after the third dose (Day 126; approximately 18 weeks of age). Serum was separated and stored at ≤−20 °C with periodic temperature monitoring until the sample was sent to the central laboratory.

Anti-hepatitis B surface antibody (anti-HBs) concentrations were measured at a central laboratory (Oncquest Laboratories Ltd., Hyderabad, India) using a validated quantitative chemiluminescent microparticle immunoassay (CMIA) (Abbott ARCHITECT CMIA kit). The assay was performed with appropriate quality controls and calibration against the WHO International Standard for anti-HBs. Values are expressed in milli-international units per millilitre (mIU/mL).

### 2.7. Immunogenicity Endpoints

The primary immunogenicity endpoint was the seroprotection rate at Day 126, defined as the proportion of participants with anti-HBs concentrations ≥10 mIU/mL, a threshold widely accepted as protective against HBV infection [4,5]. Secondary immunogenicity endpoints included: geometric mean concentration (GMC) of anti-HBs at Day 126; geometric mean fold rise (GMFR) in anti-HBs from baseline to Day 126; and proportion of participants with a significant antibody response, defined as a ≥2-fold and ≥4-fold rise from baseline.

### 2.8. Safety Assessments

All participants who received at least one dose of the study vaccine were included in the safety evaluation. Following each vaccination, participants were observed for at least 60 min at the study site for any immediate adverse reactions.

During each vaccination visit, the parent(s) or legally acceptable representative (LAR) received a structured diary card to document any solicited local or systemic adverse events (AEs) occurring within the first 7 days post-vaccination. Solicited local reactions included injection-site pain/tenderness, erythema, and swelling. Solicited systemic reactions included fever (axillary temperature ≥ 38.0 °C), irritability/fussiness, drowsiness (somnolence), and decreased appetite.

In addition to solicited AEs, all unsolicited adverse events defined as any untoward medical occurrence not prespecified as a solicited reaction were collected from the time of vaccination through 28 days after each dose using diary cards that included an open-text section for recording any other symptoms/medical events and through standardized questioning by study staff at scheduled visit time points to ascertain intercurrent illness, medical consultations, or new symptoms. Serious adverse events (SAEs) were recorded throughout the study duration. The intensity of each adverse event was graded using protocol-defined criteria, including the Common Terminology Criteria for Adverse Events (CTCAE), version 5.0, and the Division of AIDS (DAIDS) Table for Grading the Severity of Adult and Pediatric Adverse Events, version 2.0. For febrile events, severity was classified according to the Brighton Collaboration case definition scale. Causality assessment was performed by the study investigator, who determined the relationship between each reported event and the study vaccine.

To minimize reporting bias in parent-/LAR-reported safety outcomes, parents/LARs remained blinded to vaccine allocation and were instructed to report any medical events occurring during the study period irrespective of perceived relatedness to vaccination. Solicited events were collected using a standardized, structured diary card with predefined terms, time windows, and grading instructions. Parents/LARs were trained by site staff at each vaccination visit on how to record solicited symptoms, including use of a thermometer for temperature recording and, where applicable, measurement of injection-site erythema and swelling. Diary cards were reviewed by study staff at subsequent visits to ensure completeness and consistent interpretation, and any unclear entries were clarified with the parent/LAR using prespecified definitions.

### 2.9. Statistical Analysis

The primary hypothesis was that BEVAC^®^ is non-inferior to GeneVac-B^®^ with respect to seroprotection rate following completion of the three-dose primary series. Non-inferiority was assessed using the absolute difference in seroprotection proportions (BEVAC^®^ minus comparator), with a prespecified non-inferiority margin of −10%, consistent with commonly accepted approaches in vaccine non-inferiority trials. The −10% margin was prespecified based on WHO guidance for comparative immunogenicity trials [13]. This is also consistent with commonly used immunobridging success criteria that apply a 10% margin for difference in seroprotection rates [14]. Similar −10% margins have been used in prior Hep-B-containing infant vaccine non-inferiority studies evaluating hepatitis B seroprotection [15].

Sample size calculations assumed seroprotection rates of approximately ≥95% in both groups. A sample size of ~200 infants per group was estimated to provide >90% power to demonstrate non-inferiority at a one-sided α of 0.025. To account for potential dropouts, the enrolment target was set to ~460–470 infants (~230 per group).

All data were captured and validated using clinical data management procedures, including double data entry and verification. All demographic and baseline characteristics for each treatment group were summarized for all randomized participants, defined as the intention-to-treat (ITT) population. Demographics and baseline characteristics were analysed by summary statistics. For continuous variables, n, mean and standard deviation were presented. For categorical data, frequencies were computed.

Immunogenicity analyses were performed in the per-protocol (PP) population, defined as participants who received all three doses within protocol-defined windows, had evaluable immunogenicity results, and had no major protocol deviations affecting immunogenicity assessment. For the primary endpoint, seroprotection rates at Day 126 were summarized by group with two-sided 95% confidence intervals (CIs) calculated using the Clopper–Pearson exact method. The between-group difference in seroprotection rates (BEVAC^®^ minus comparator) and its two-sided 95% CI were calculated using the Farrington–Manning method. Non-inferiority was concluded if the lower bound of the Farrington–Manning two-sided 95% CI for the difference was greater than −10%.

Secondary immunogenicity endpoints were summarized descriptively. Anti-HBs concentrations were log-transformed prior to analysis; GMCs were calculated as the antilog of the mean of log-transformed concentrations. The anti-HBs GMC ratio (test vaccine/comparator vaccine) was calculated with a 95% CI. GMFRs were derived from baseline and post-vaccination GMCs. Where applicable, between-group ratios and corresponding CIs were presented. Although no formal hypothesis testing was prespecified for secondary immunogenicity endpoints, exploratory between-group comparisons were performed. For continuous endpoints, comparisons used a two-sample Student’s *t*-test on log-transformed values with back-transformation to geometric mean ratios and two-sided 95% CI. For categorical endpoints, Pearson’s chi-square or Fisher’s exact tests were used as appropriate; risk differences (test–comparator) with 95% CIs were estimated. *p*-values are nominal and interpreted exploratorily; no multiplicity adjustment was applied. No imputation was performed for missing immunogenicity data.

Safety analyses were performed in the safety population, defined as all participants who received at least one dose of the study vaccine. All reported adverse events during the entire study period were summarized descriptively by calculating frequencies and were listed per subject, including severity and relationship to the vaccine (causality). The number and percentage of participants with AEs were presented by system organ class (SOC) and preferred term (PT). Two-sided 95% exact confidence intervals (CIs) were calculated for occurrence rates of reported AEs and SAEs during the study. All AEs were coded using the Medical Dictionary for Regulatory Activities (MedDRA™; version 25.1) coding dictionary.

All analyses were conducted using SAS^®^ 9.4 software (SAS Institute Inc., Cary, NC, USA).

### 2.10. Data Management and Independent Statistical Oversight

Clinical data management and database validation were performed by an external data management vendor in accordance with a predefined data management plan. The database was locked prior to analysis. Statistical analyses were performed by an independent statistician (not employed by the sponsor) using the locked dataset and the predefined statistical analysis plan. The sponsor and study team reviewed the results for clinical interpretation; however, data handling, database lock and statistical analyses were performed independently to support objectivity.

## 3. Results

### 3.1. Participant Disposition and Analysis Populations

**Figure 1:**
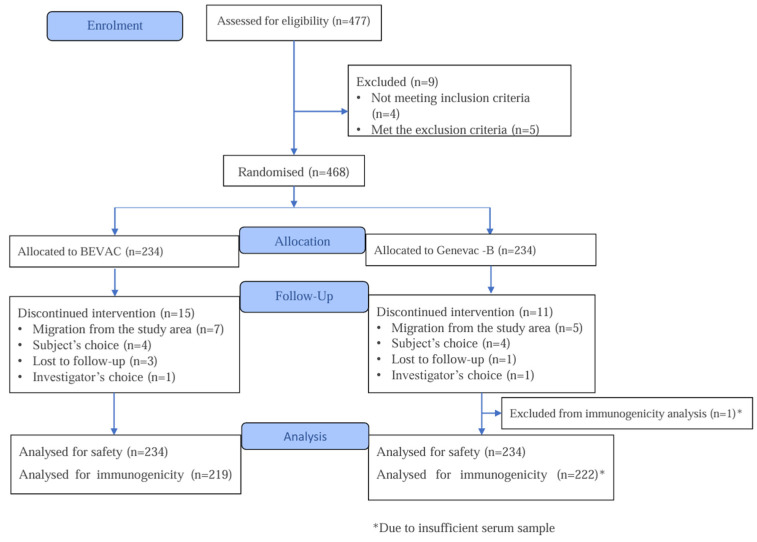
CONSORT flow diagram of participant enrolment, allocation, follow-up, and analysis populations.

Between September 2023 and February 2024, 477 newborns were screened across seven study sites; 468 met eligibility criteria and were randomized (BEVAC^®^, n = 234; GeneVac-B^®^, n = 234). Twenty-six participants (15 in the BEVAC^®^ group and 11 in GeneVac-B^®^) did not complete the study due to withdrawal of consent (four in each group), loss to follow-up (three and one, respectively), migration from the study area (seven and five, respectively), or investigator’s choice due to COVID-19 infection in the parents (one in each group). In addition, one participant in the comparator group completed the study but was excluded from the PP population due to insufficient serum sample for immunogenicity assessments. The PP immunogenicity population comprised 441 infants (219 in BEVAC^®^ and 222 in GeneVac-B^®^). The safety population included all randomized participants who received at least one vaccine dose (n = 468). Participant disposition is shown in Figure 1 (CONSORT diagram).

### 3.2. Baseline Demographic and Clinical Characteristics

Baseline characteristics were comparable between the groups (Table 1). In the BEVAC^®^ and GeneVac-B^®^ arms, 40.2% and 47.9% of participants were female, respectively. Mean (±SD) birth weight was 2.9 ± 0.32 kg in the BEVAC^®^ group and 2.9 ± 0.33 kg in the GeneVac-B^®^ group. Mean gestational age was approximately 39 weeks in both groups. Approximately 98% of deliveries occurred in hospitals and ~45% were caesarean deliveries, with no meaningful between-group differences.

Maternal antenatal HBsAg results were available for approximately 90% of mothers; none were reported as HBsAg-positive, consistent with the protocol exclusion criteria. A small proportion of infants had detectable baseline anti-HBs antibodies (BEVAC^®^, 5.1%; GeneVac-B^®^, 5.6%), consistent with maternally derived antibodies. Baseline anti-HBs GMCs were similar between groups (6.8 mIU/mL in both arms).

### 3.3. Immunogenicity

#### 3.3.1. Seroprotection Rate (Primary Endpoint)

**Figure 2:**
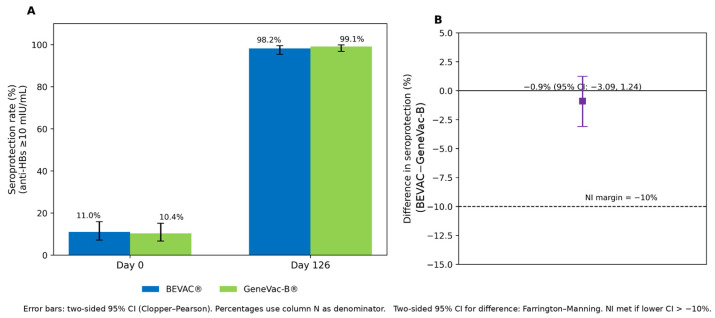
**Comparison of seroprotection rates and demonstration of non-inferiority.** (**A**). Seroprotection rates at Day 0 and Day 126 for BEVAC^®^ vs. GeneVac-B^®^, with 95% CI in PP population (N = 441); (**B**). Forest plot for non-inferiority analysis by percentage differences in seroprotection rates at Day 126 (test minus control) with 95% CI.

The seroprotection rates at Day 0 and Day 126 for both the vaccines with non-inferiority assessment are included in Figure 2A,B. At Day 126 (28 days after dose 3), seroprotection (anti-HBs ≥10 mIU/mL) was observed in 98.2% (215/219) of infants in the BEVAC^®^ group and 99.1% (220/222) in the GeneVac-B^®^ group (PP population) (Figure 2A). The absolute difference in seroprotection rates (BEVAC^®^ minus GeneVac-B^®^) was −0.9%, with a two-sided 95% CI of −3.09% to 1.24%. As the lower bound of the CI was above the prespecified non-inferiority margin of −10%, non-inferiority was concluded (Figure 2B).

#### 3.3.2. Anti-HBs Antibody Concentrations

The magnitude of immune response (anti-HBs GMCs, GMFR, and fold rise) is presented in Table 2. At baseline, anti-HBs concentrations were low and similar between groups (GMC: 6.78 mIU/mL for BEVAC^®^ and 6.77 mIU/mL for GeneVac-B^®^). At Day 126, both vaccines induced robust antibody responses. The Day 126 anti-HBs GMC was 282.78 mIU/mL (95% CI: 225.16–355.16) in the BEVAC^®^ group and 366.22 mIU/mL (95% CI: 294.40–455.57) in the GeneVac-B^®^ group. The GMFR from baseline to Day 126 was 41.69 (95% CI: 31.79–54.67) for BEVAC^®^ and 54.06 (95% CI: 41.86–69.82) for GeneVac-B^®^. At Day 126, anti-HBs ≥100 mIU/mL was observed in approximately 84% of BEVAC^®^ recipients and 86% of GeneVac-B^®^ recipients, indicating high-level responses in both groups.

In an exploratory between-group comparison using a two-sample Student’s *t*-test on log-transformed Day 126 concentrations, there was no statistically significant difference between vaccines; the geometric mean concentration ratio (BEVAC/GeneVac-B) was 0.77 (95% CI: 0.53, 1.12; *p* = 0.1765). The GMFR ratio (BEVAC/GeneVac) was 0.77 (95% CI: 0.53, 1.12; *p* = 0.1701). The proportions achieving a ≥2-fold rise were 97.7% in both groups (*p* = 1.00, Fisher’s exact test), and a ≥4-fold rise was observed in 84.9% vs. 90.1% of participants (*p* = 0.101, chi-square test). At Day 126, anti-HBs ≥100 mIU/mL was observed in 84.02% (95% CI: 78.48, 88.61) of BEVAC recipients and 86.03% (95% CI: 80.77, 90.31) of GeneVac-B recipients (*p* = 0.553, chi-square test), indicating high-level response in both groups.

### 3.4. Safety and Reactogenicity

#### 3.4.1. Overall Safety

**Figure 3:**
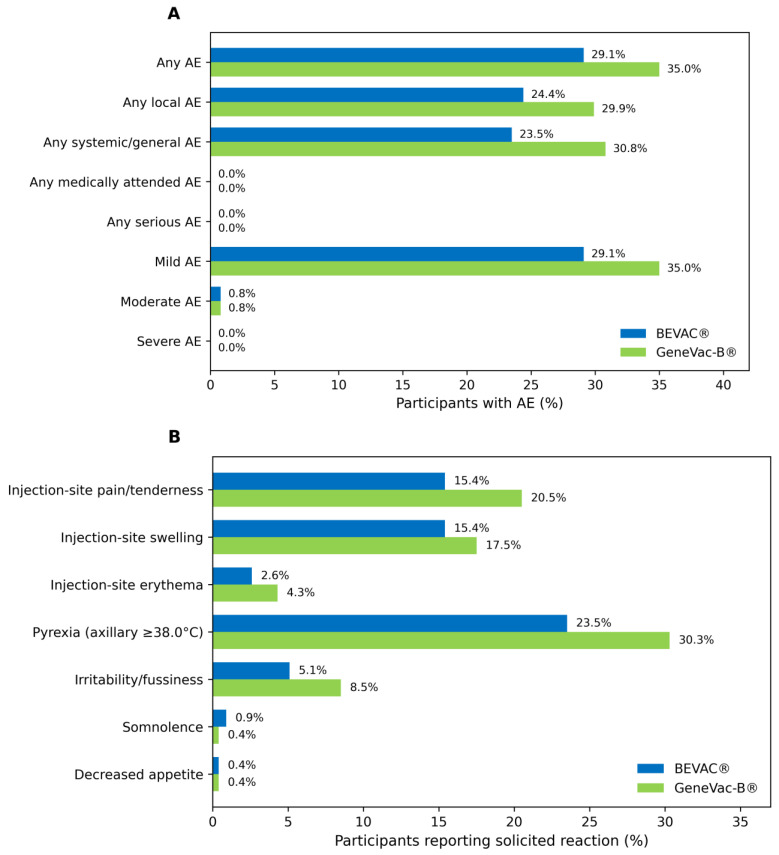
**Comparison of overall safety and reactogenicity and solicited adverse events.** (**A**) Overall summary of safety and reactogenicity, reported during entire study period (Day 0 to Day 126); (**B**) Proportion of participants reporting solicited (local and systemic) adverse events occurring between Day 0 and Day 6 after each dose of vaccination (n = 468).

Over the entire study period, 148 AEs were reported among 69 participants (29.06%) in the BEVAC^®^ cohort versus 193 AEs in 82 participants (35.04%) in the GeneVac-B^®^ Cohort (Figure 3A). All the reported events were solicited. There were no serious AEs or deaths reported. No participant was withdrawn due to an adverse event. No medically attended adverse events attributable to study vaccination were reported. Most AEs were mild in intensity; moderate events were uncommon and were reported by 0.8% of participants in each group, and no severe or life-threatening events were reported (Figure 3A).

#### 3.4.2. Solicited Local and Systemic Reactions

Solicited adverse events within 7 days after vaccination were reported at similar rates in both groups (Figure 3B). Across all doses, 29.06% of infants in the BEVAC^®^ group and 35.04% in the GeneVac-B^®^ group experienced at least one solicited reaction. Most reactions occurred within 1–2 days after vaccination and resolved without sequelae.

Fever was the most frequently reported solicited systemic event, occurring in 23.5% of BEVAC^®^ recipients and 30.3% of GeneVac-B^®^ recipients. Fever episodes were predominantly mild (<38.5 °C) and resolved within 1–2 days; no severe fever event was reported. The most common solicited local reactions were injection-site pain/tenderness and swelling. Injection-site pain/tenderness occurred in 15.4% of BEVAC^®^ recipients and 20.5% of GeneVac-B^®^ recipients. Injection-site swelling occurred in 15.4% and 17.5%, respectively, while erythema was uncommon (2.6% vs. 4.3%).

Among systemic reactions besides fever, irritability (fussiness) was noted in 5.1% vs. 8.5% of infants (BEVAC^®^ vs. comparator), drowsiness (somnolence) in 0.9% vs. 0.4%, and decreased appetite in 0.4% vs. 0.4%, respectively. Detailed severity distribution and timing by dose are provided in the Appendix A (Table A1, Table A2 and Table A3). Table A1 summarizes all reported AEs by system organ class (SOC) and preferred term (PT), including maximum severity grade for each event; events were predominantly mild (Grade 1), moderate (Grade 2) events were uncommon, and no severe or life-threatening events (Grade 3/4) were reported. Table A2 summarizes the overall safety comparison and comparison by risk period. Table A3 provides additional details for fever, including severity grading and timing (onset and duration). Fever >100.4 °F occurred in 22.22% (52/234; 95% CI: 17.06–28.10) of BEVAC^®^ recipients and 26.07% (61/234; 95% CI: 20.57–32.19) of GeneVac-B^®^ recipients (Table A3). Most fever episodes were Grade 1 (21.79% vs. 25.21%), Grade 2 events were rare (0.43% vs. 0.85%), and no Grade 3/4 fever occurred. Fever typically began on Day 2 (BEVAC^®^) or Day 3 (GeneVac-B^®^) and resolved within 2 days in both groups; temperatures ranged from 100.7 to 102.5 °F and 100.5 to 102.8 °F, with mean peak temperatures of 101.5 °F (95% CI: 101.40–101.70) and 101.9 °F (95% CI: 101.75–102.06), respectively.

#### 3.4.3. Unsolicited Adverse Events

There were no unsolicited adverse events reported in the study.

#### 3.4.4. Serious Adverse Events

No SAEs were reported in either group during the active study period (birth to ~18 weeks).

## 4. Discussion

This multicentre phase IV, randomized, active-controlled non-inferiority study showed that Biological E’s monovalent recombinant hepatitis B vaccine (BEVAC^®^) is non-inferior to GeneVac-B^®^ when given to term neonates at birth and at 6 and 14 weeks of age. Seroprotection one month after the third dose was high in both groups, with more than 98% of infants achieving anti-HBs concentrations at or above the accepted protective threshold. These findings are in line with the consistently strong performance of recombinant hepatitis B vaccines in immunocompetent infants and with expectations set out in major guidance and reference sources [8,16].

The non-inferiority conclusion was supported by the between-group confidence interval for seroprotection. The lower bound remained above the prespecified margin of minus 10%, which is consistent with commonly used approaches for comparative vaccine immunogenicity studies. Secondary immunogenicity endpoints supported the primary endpoint findings. The geometric mean concentrations rose from baseline values of ~6 to 282 and 366 mIU/mL for the BEVAC^®^ and comparator arms, respectively. On Day 126, the geometric mean ratio with a 95% CI for the test and comparator vaccines was 0.77 (95% CI: 0.53, 1.12). While NI for GMCs was not prespecified, the observed CI lower bound exceeds the 0.5 margin used in some NI trials for hepatitis B vaccines [17]. GMFRs from baseline to post-dose 3 were high in both arms (41.69-fold for BEVAC^®^ and 54.06-fold for GeneVac-B^®^). The proportions achieving a ≥2-fold rise in anti-HBs IgG were similar between groups, and the ≥4-fold rise proportions were 84.9% and 90.1%, respectively.

The magnitude of the immune response (GMCs and GMFR) induced by the BEVAC^®^ group was numerically lower than for the comparator arm; however, the differences were not significant.

Variability in GMCs across hepatitis B vaccine products has been reported previously and may reflect differences in manufacturing processes, antigen presentation, or adjuvant formulation, even where nominal antigen content is similar [12,18,19].

The proportion of participants with anti-HBs IgG ≥ 100 mIU/mL, a criterion which is generally considered as an indicator of long-term protection against hepatitis B, was high and comparable between the groups (~84% in the BEVAC^®^ and ~86% in the GeneVac-B^®^ group, respectively) indicating such non-significant differences in GMC do not have an impact on the efficacy and long-term protection of the vaccine [20].

This trial also provides important safety data for BEVAC^®^ in neonates. Both vaccines were well tolerated, with no SAEs or deaths, and no withdrawals due to adverse events. Solicited local and systemic reactions were predominantly mild-to-moderate and consistent with the known reactogenicity profile of hepatitis B vaccines administered in infancy [12,16,18,21].

Fever was the most frequently reported systemic event, with rates within the expected range for routine infant immunizations, particularly in the context of concomitant administration of other vaccines at later visits [21]. No clinically significant safety signals were observed, including no febrile seizures, hypotonic–hyporesponsive episodes, or hypersensitivity reactions.

Although multiple studies of infant hepatitis B vaccines are available, this phase IV multicentre head-to-head trial provides timely, program-relevant evidence by directly comparing BEVAC^®^ with a licensed comparator on the routine Indian schedule, including a birth dose within 24 h. Much of the published comparative infant literature is more than a decade old, and because vaccine manufacturing processes may evolve over time (e.g., scale-up or process optimization) within established comparability frameworks, contemporary clinical data remain valuable to confirm consistent immunogenicity and tolerability under current conditions. Conduct across seven sites enhances generalizability to routine practice, and the demonstration of non-inferior seroprotection with a comparable safety profile supports confidence in BEVAC^®^ for broader use following WHO prequalification.

This study has limitations. First, immunogenicity was assessed at 28 days following completion of the primary series (Day 126), and long-term antibody persistence was not evaluated. Because anti-HBs concentrations and seroprotection rates are known to decline over time after infant hepatitis B vaccination, the Day 126 GMCs reported here should be interpreted as short-term post-series response and may not reflect antibody levels at later time points. Kumar D, et al. reported an age-related decline in protective anti-HBs antibodies, with approximately 56–63% of vaccinated Indian children aged 5–9 years having protective titres and 40% of those aged 9–10 years remaining above the protective threshold [22]. The clinical implications of declining anti-HBs titres in otherwise healthy vaccinees are not fully defined. Importantly, protection in immunocompetent individuals is considered to depend largely on immune memory, and an anamnestic response is known to occur even when circulating anti-HBs concentrations fall below the minimum protection threshold [23,24]. Accordingly, accumulated data suggest that the booster doses are generally unnecessary in immunocompetent individuals who complete a primary series [19]. Therefore, longer follow-up with additional time points (e.g., 1–5 years) and/or assessment of an anamnestic response (after a challenge dose) would strengthen the evidence on antibody persistence and durability of protection [23,24]. Second, infants born to HBsAg-positive mothers were excluded; therefore, the findings apply to routine immunization of infants not requiring HBIG and do not directly address prevention of MTCT in high-risk newborns. Infants born to HBs Ag-positive mothers, particularly when maternal HBV DNA levels are high (often associated with HBeAg positivity), have substantially higher perinatal exposure and represent the group at greatest risk for MTCT. In this setting, prevention strategies rely not only on vaccine immunogenicity but also on timely programmatic interventions and, in selected cases, maternal antiviral prophylaxis [3,5]. MTCT prevention programs include administration of the hepatitis B vaccine plus HBIG soon after birth, preferably within 12 h of birth, followed by completion of the series and post-vaccination serologic testing [5]. In addition, maternal antiviral prophylaxis during pregnancy is recommended in some settings to further reduce MTCT risk [3]. Accordingly, immunogenicity studies of standard routine immunization settings should not be extrapolated to infer effectiveness against MTCT prevention among infants born to HBsAg-positive mothers; dedicated studies in this population, assessed under standard MTCT interventions and including infection outcomes (e.g., infant HBsAg and/or HBV DNA), are needed. Third, the study was single-blind due to practical constraints related to vaccine presentation. Bias in immunogenicity outcomes is unlikely given objective laboratory endpoints; while reactogenicity is parent-reported, the observed rates and patterns were consistent with established vaccine profiles.

In summary, BEVAC^®^ administered at birth and at 6 and 14 weeks elicited high seroprotection rates and robust anti-HBs responses, with a safety profile comparable to a licensed comparator vaccine. These findings support BEVAC^®^ as an effective and well-tolerated option for hepatitis B prevention in neonates and infants.

## 5. Conclusions

In this multicentre phase IV non-inferiority trial in term neonates, BEVAC^®^ showed non-inferior seroprotection compared with a comparator (GeneVac-B^®^) following a 0–6–14-week schedule. Both vaccines induced high seroprotection rates and robust anti-HBs concentrations, and were well tolerated, with no serious adverse events. These results affirm that BEVAC^®^ is a safe and effective option for neonatal hepatitis B immunization. The availability of this vaccine, now WHO-prequalified, can bolster hepatitis B control efforts by improving access to affordable vaccines for newborns. Widespread use of BEVAC^®^ in infant immunization programs could help sustain high protection rates and contribute to the global elimination of HBV transmission in the long term.

## Figures and Tables

**Table 1:** Baseline characteristics of the study population.

Characteristic	BEVAC Group (N = 234)	GeneVac-B Group (N = 234)
Male—n (%)	140 (59.8%)	122 (52.1%)
Female—n (%)	94 (40.2%)	112 (47.9%)
Birth weight (kg)—m ± SD	2.9 ± 0.32	2.9 ± 0.33
Gestational age (weeks)—m	38.9	39.1
Vaginal delivery—n (%)	130 (55.6%)	128 (54.7%)
Caesarean delivery—n (%)	104 (44.4%)	106 (45.3%)
Baseline anti-HBs ≥10 mIU/mL—n (%)	12 (5.1%)	13 (5.6%)
Baseline anti-HBs GMC (mIU/mL) ^a^	6.8	6.8

m: mean; SD: standard deviation. ^a^ Geometric mean concentration (GMC) of anti-HBs at baseline. The lower limit of quantitation was 2 mIU/mL. GMC calculation includes all infants; those with undetectable anti-HBs were assigned a value of 1 mIU/mL for computation.

**Table 2:** Immunogenicity results (GMCs, GMFR, and fold rise) at 28 days after the third dose (Day 126).

Anti-HBs IgG Concentration Endpoint	BEVAC (N = 219)	GeneVac-B (N = 222)	Between-Group Estimate (BEVAC vs. GeneVac-B)	*p*-Value
GMC [95% CI] at pre-vaccination (Day 0)	6.78 [6.08, 7.57]	6.77 [6.00, 7.65]	-	-
GMC [95% CI] at post-vaccination (Day 126)	282.78 [225.16, 355.16]	366.22 [294.40, 455.57]	GMR = 0.77 [0.53, 1.12]	0.1765
Geometric mean fold rise [95% CI] (Day 126 from baseline)	41.69 [31.79, 54.67]	54.06 [41.86, 69.82]	GMFR ratio = 0.77 [0.53, 1.12]	0.1701
≥2-fold rise, n (%) [95% CI]	97.7% [94.75, 99.25]	97.7% [94.82, 99.26]	Risk diff = 0.0% [−4.3%, 4.2%]	1.000
≥4-fold rise, n (%) [95% CI]	84.9% [79.49, 89.39]	90.1% [85.38, 93.68]	Risk diff = −5.2% [−13.8%, 3.6%]	0.101
Anti-HBs ≥100 mIU/mL at Day 126, n (%) [95% CI]	84.02% [78.48, 88.61]	86.03% [80.77, 90.31]	Risk diff = −2.02% [−8.68%, 4.64%]	0.553

***Note:** N: Number of participants with immunogenicity data; GMC = geometric mean concentration; GMFR = geometric mean fold rise; CI: confidence interval*.

## Data Availability

All relevant data are presented in this paper. Any additional data will be available upon reasonable request.

## Appendix A. Full Inclusion and Exclusion Criteria

Inclusion Criteria:

- All subjects were expected to satisfy ALL the following criteria at study entry:
- Subjects’ parent(s)/LAR(s) who, in the opinion of the investigator, can and will comply with the requirements of the protocol (e.g., willingness to allow audio–visual recording of the consent process, completion of the diary cards, return for follow-up visits and willingness to allow blood sampling).
- Written or thumb-printed informed consent obtained from the parent(s)/LAR(s) of the subject prior to performing any study-specific procedure.
- A male or female child, of less than 24 h of age at the time of vaccination, without prior immunization of hepatitis B vaccine (birth dose).
- Born full-term (i.e., after a gestation period of at least 37 weeks) with a birth weight of no less than 2500 g (i.e., ≥2500 gms).
- Mother is seronegative (laboratory-confirmed) for HBsAg prior to the birth of the child.
- Healthy subjects, as established by medical history and clinical examination before entering into the study.
- Exclusion Criteria:
- The following criteria were checked at the time of study entry. If ANY exclusion criterion applied, the subject was not to be included in the study:
- Child in care:
- *[A child who has been placed under the control or protection of an agency, organization, institution or entity by the courts, the government, or a government body, acting in accordance with powers conferred on them by law or regulation. The definition of a child in care can include a child cared for by foster parents or living in a care home or institution, provided that the arrangement falls within the definition above. The definition of a child in care does not include a child who is adopted or has an appointed legal guardian.]*
- Use of any investigational or non-registered product (drug or vaccine) other than the study vaccine during the first 24 h period from birth, before the administration of the study vaccine, or planned use during the study period.
- Any medical condition that in the judgment of the investigator would make intramuscular injection unsafe.
- Any confirmed or suspected immunosuppressive or immunodeficient condition, based on medical history and physical examination (no laboratory testing required).
- Prior immunization with hepatitis B vaccine birth dose with the exception of BCG and/or oral/injectable polio vaccine.
- Planned administration of immunoglobulins and/or any blood products before the administration of the study vaccine or during the study period.
- Family history of congenital or hereditary immunodeficiency.
- Major congenital defects (defined as major malformations causing significant functional or cosmetic impairment or being life-limiting).
- Acute illness and/or fever at the time of vaccination.
- Fever is defined as the endogenous elevation of at least one measured body temperature of ≥38 °C (≥100.4 °F).
- Acute or chronic, clinically significant pulmonary, cardiovascular, hepatic or renal functional abnormality, as determined by physical examination.
- Administration of long-acting immune-modifying drugs at any time during the study period.
- Planned or elective surgery during the course of the study.
- Any criteria which, in the opinion of the investigator, suggest that the subject would not be compliant with the study protocol.

**Table A1:** **Summary of AEs by SOC and PT by severity—safety population (N = 468)**.

SOC/PT/Severity, N1 (%) [95% CI] n	BEVAC (N = 234)	GENEVAC-B (N = 234)
OVERALL	68 (29.1%) [23.33:35.33] 148	82 (35.0%) [28.94:41.53] 193
**General Disorders And Administration Site Conditions**	**68 (29.1%) [23.33:35.33]145**	**82 (35.0%) [28.94:41.53]191**
**Injection-Site Erythema**	**6 (2.6%) [0.95:5.50]6**	**10 (4.3%) [2.07:7.72]10**
Mild	5 (2.1%) [0.70:4.92]5	10 (4.3%) [2.07:7.72]10
Moderate	1 (0.4%) [0.01:2.36]1	0 (0.0%) [NE]0
Severe	0 (0.0%) [NE]0	0 (0.0%) [NE]0
Life-threatening	0 (0.0%) [NE]0	0 (0.0%) [NE]0
**Injection-Site Pain**	**36 (15.4%) [11.01:20.66]36**	**48 (20.5%) [15.53:26.26]48**
None	0 (0.0%) [NE]0	0 (0.0%) [NE]0
Mild	36 (15.4%) [11.01:20.66]36	48 (20.5%) [15.53:26.26]48
Moderate	0 (0.0%) [NE]0	0 (0.0%) [NE]0
Severe	0 (0.0%) [NE]0	0 (0.0%) [NE]0
Life-threatening	0 (0.0%) [NE]0	0 (0.0%) [NE]0
**Injection-Site Swelling**	**36 (15.4%) [11.01:20.66]36**	**41 (17.5%) [12.88:23.01]41**
None	0 (0.0%) [NE]0	0 (0.0%) [NE]0
Mild	36 (15.4%) [11.01:20.66]36	41 (17.5%) [12.88:23.01]41
Moderate	0 (0.0%) [NE]0	0 (0.0%) [NE]0
Severe	0 (0.0%) [NE]0	0 (0.0%) [NE]0
Life-threatening	0 (0.0%) [NE]0	0 (0.0%) [NE]0
**Post-vaccinal Irritability**	**12 (5.1%) [2.68:8.79]12**	**20 (8.5%) [5.30:12.89]20**
None	0 (0.0%) [NE]0	0 (0.0%) [NE]0
Mild	12 (5.1%) [2.68:8.79]12	20 (8.5%) [5.30:12.89]20
Moderate	0 (0.0%) [NE]0	0 (0.0%) [NE]0
Severe	0 (0.0%) [NE]0	0 (0.0%) [NE]0
Life-threatening	0 (0.0%) [NE]0	0 (0.0%) [NE]0
**Pyrexia**	**55 (23.5%) [18.22:29.47]55**	**71 (30.3%) [24.52:36.67]72**
None	0 (0.0%) [NE]0	0 (0.0%) [NE]0
Mild	54 (23.1%) [17.84:29.01]54	69 (29.5%) [23.72:35.78]70
Moderate	1 (0.4%) [0.01:2.36]1	2 (0.9%) [0.10:3.05]2
Severe	0 (0.0%) [NE]0	0 (0.0%) [NE]0
Life-threatening	0 (0.0%) [NE]0	0 (0.0%) [NE]0
**Metabolism And Nutrition Disorders**	**1 (0.4%) [0.01:2.36]1**	**1 (0.4%) [0.01:2.36]1**
Decreased Appetite	1 (0.4%) [0.01:2.36]1	1 (0.4%) [0.01:2.36]1
None	0 (0.0%) [NE]0	0 (0.0%) [NE]0
Mild	1 (0.4%) [0.01:2.36]1	1 (0.4%) [0.01:2.36]1
Moderate	0 (0.0%) [NE]0	0 (0.0%) [NE]0
Severe	0 (0.0%) [NE]0	0 (0.0%) [NE]0
Life-threatening	0 (0.0%) [NE]0	0 (0.0%) [NE]0
**Nervous System Disorders**	**2 (0.9%) [0.10:3.05]2**	**1 (0.4%) [0.01:2.36]1**
Somnolence	2 (0.9%) [0.10:3.05]2	1 (0.4%) [0.01:2.36]1
None	0 (0.0%) [NE]0	0 (0.0%) [NE]0
Mild	2 (0.9%) [0.10:3.05]2	1 (0.4%) [0.01:2.36]1
Moderate	0 (0.0%) [NE]0	0 (0.0%) [NE]0
Severe	0 (0.0%) [NE]0	0 (0.0%) [NE]0
Life-threatening	0 (0.0%) [NE]0	0 (0.0%) [NE]0

**Note:** Percentages were calculated using the column header count as the denominator. The 95% CIs were calculated by the Clopper–Pearson method. N1: subject count, N: sample size, n: event count. General Note: All AEs are represented as subject count (percentage of subjects) [95% CI] event count.

**Table A2:** Summary of overall adverse events and adverse events by risk period.

Category, N1 (%) [95% CI] n	BEVAC (N = 234)	GENEVAC-B (N = 234)
Any AE	68 (29.06%) [23.33:35.33] [148]	82 (35.04%) [28.94:41.53] [193]
Any Serious AE	0 (0.00%) [NE] [0]	0 (0.00%) [NE] [0]
Any Local AE	57 (24.36%) [19.00:30.38] [78]	70 (29.91%) [24.12:36.22] [99]
Any Systemic/General AE	55 (23.50%) [18.22:29.47] [70]	72 (30.77%) [24.92:37.11] [94]
Any Medically Attended AE	0 (0.00%) [NE] [0]	0 (0.00%) [NE] [0]
Any Grade 3 AE	0 (0.00%) [NE] [0]	0 (0.00%) [NE] [0]
Any Solicited AE	68 (29.06%) [23.33:35.33] [148]	82 (35.04%) [28.94:41.53] [193]
Any Unsolicited AE	0 (0.00%) [NE] [0]	0 (0.00%) [NE] [0]
AEs in Risk Period 1 (Day 0 to Day 42)	58 (24.79%) [19.39:30.83] [138]	68 (29.06%) [23.33:35.33] [174]
AEs in Risk Period 2 (Day 42 to Day 98)	6 (2.56%) [0.95:5.50] [6]	10 (4.27%) [2.07:7.72] [13]
AEs in Risk Period 3 (Day 98 to Day 126)	4 (1.71%) [0.47:4.32] [4]	6 (2.56%) [0.95:5.50] [6]

**Note:** Percentages were calculated using the column header count as the denominator. The 95% CIs were calculated by the Clopper–Pearson method. N1: subject count, N: sample size, n: event count. General Note: All AEs are represented as subject count (percentage of subjects) [95% CI] event count. Solicited local and general AEs were recorded for 7 days (Day 0–Day 6) after the vaccination. Unsolicited adverse events reported after each dose of vaccination. SAEs were recorded after vaccination up to the end of the study (visit 4).

**Table A3:** Summary of fever after vaccination (Day 0–Day 6)—safety population (N = 468).

Parameter N1 (%) [CI] n	BEVAC (N = 234)	GENEVAC-B (N = 234)
Number of subjects whose temperature is >100.4 °F	52 (22.22%) [17.06:28.10]52	61 (26.07%) [20.57:32.19]61
Lasting less than 24 h	5 (2.14%) [0.70:4.92]5	2 (0.85%) [0.10:3.05]2
Lasting more than 24 h	47 (20.09%) [15.15:25.80]47	59 (25.21%) [19.78:31.28]59
Grade 1	51 (21.79%) [16.68:27.64]51	59 (25.21%) [19.78:31.28]59
Grade 2	1 (0.43%) [0.01:2.36]1	2 (0.85%) [0.10:3.05]2
Grade 3	0 (0.00%) [NE]0	0 (0.00%) [NE]0
Grade 4	0 (0.00%) [NE]0	0 (0.00%) [NE]0
Temperature recorded (min:max)	(100.7:102.5)	(100.5:102.8)
Mean temperature (with 95% CI)	101.5 (101.40:101.70)	101.9 (101.75:102.06)
Time to onset (days)	(2 Days)	(3 Days)
Time to resolution (days)	(2 Days)	(2 Days)

**Note:** Percentages were calculated using the column header count as the denominator. The 95% CIs were calculated by the Clopper–Pearson method. N1: subject count, N: sample size, n: event count.
